# Lactoferrin Ameliorates Dry Eye Disease Potentially through Enhancement of Short-Chain Fatty Acid Production by Gut Microbiota in Mice

**DOI:** 10.3390/ijms222212384

**Published:** 2021-11-17

**Authors:** Samuel Connell, Motoko Kawashima, Shigeru Nakamura, Toshihiro Imada, Hiromitsu Yamamoto, Kazuo Tsubota, Shinji Fukuda

**Affiliations:** 1 Department of Ophthalmology, Keio University School of Medicine, Shinjuku-ku, Tokyo 160-8582, Japan; Samuelconnell.sc@gmail.com (S.C.); motoko326@gmail.com (M.K.); vdtwork@gmail.com (S.N.); fd3sima@gmail.com (T.I.); 2 Institute for Advanced Biosciences, Keio University, Tsuruoka 997-0052, Yamagata, Japan; yamahiro@ttck.keio.ac.jp; 3 Tsubota Laboratory, Inc., Tokyo 160-0016, Japan; 4 Transborder Medical Research Center, University of Tsukuba, Tsukuba 305-8575, Ibaraki, Japan; 5 Gut Environmental Design Group, Kanagawa Institute of Industrial Science and Technology, Kawasaki 210-0821, Kanagawa, Japan

**Keywords:** lactoferrin, lacrimal gland, dry eye, gut microbiota, short-chain fatty acid

## Abstract

Lactoferrin is a glycoprotein found at high concentrations within exocrine secretions, including tears. Low levels of lactoferrin have been implicated in the loss of tear secretion and ageing. Furthermore, lactoferrin possesses a range of functionalities, including anti-inflammatory properties and the ability to modulate the gut microbiota. Expanding evidence demonstrates a crucial role of the gut microbiota in immune regulation and development. The specific composition of bacterial species of the gut has a profound influence on local and systemic inflammation, leading to a protective capacity against a number of inflammatory diseases, potentially by the induction of regulatory immune cells. In this study, we demonstrated that oral administration of lactoferrin maintains tear secretion in a restraint and desiccating stress induced mouse model of dry eye disease. Furthermore, we revealed that lactoferrin induces the reduction of inflammatory cytokines, modulates gut microbiota, and induces short-chain fatty acid production. Whereas, the antibiotic vancomycin abrogates the effects of lactoferrin on dry eye disease and significantly reduces short-chain fatty acid concentrations. Therefore, this protective effect of LF against a mice model of DED may be explained by our observations of an altered gut microbiota and an enhanced production of immunomodulatory short-chain fatty acids.

## 1. Introduction

Dry eye disease (DED) is an extremely prevalent multifactorial disease of ageing, characterised by a significant reduction in tear secretion [1]. The pathogenesis of the disease is linked to both ageing and excessive inflammation of the lacrimal gland (LG), leading to the loss of function of secretory acini cells [1]. Furthermore, DED symptoms can have extremely detrimental effects on both the quality of life and productivity of sufferers [2]. Epidemiological studies have demonstrated that DED predominantly affects the elderly due to age-related loss of LG function [1,3,4], with between 14.6% [3] and 33.7% [4] of those over 65 years old presenting with symptoms. Additionally, due to an increasingly aged population in developed nations, the disease burden will become significantly worse in the forthcoming decades. It is thought that the age-related gradual decline in LG function and tear hyposecretion is caused by chronic inflammation, a result of the accumulation of reactive oxygen species (ROS), leads to cell death, DNA damage, and lipid and protein degradation [5,6,7]. Further activation of the MAPK signalling pathways consequently leads to increase cytokine production [8,9]. The subsequent tear flow insufficiency results in a vicious cycle of increased sheer stress, inflammation, and ocular surface abrasions, leading to exacerbated symptoms [8,10]. 

Lactoferrin (LF) is an 80-kDa iron binding glycoprotein and a major component of various exocrine secretions, especially colostrum and tear fluid, which present high LF concentrations [11,12]. LF was initially shown to present antimicrobial activity but has since been shown to possess a plethora of properties, including microbiota modulation, cell growth promotion, anti-tumorigenesis, and anti-inflammation [13,14,15,16,17,18,19,20,21,22,23,24,25,26,27,28,29]. Although the majority of this research has been performed in animal models, a beneficial impact of LF on human health has been demonstrated. A number of clinical trials have shown that the milk protein is of particular importance for promoting gut health in neonates [15,22,23]. However, it must be noted that due to differences in gut maturity between neonate and adults, the impact of LF may be more pronounced in the young, potentially due to increased bioavailability at the gut. Nevertheless, LF’s role in the control of viral and microbial infection has been shown in both neonate and adult patients [24,25]. Moreover, in adult patients LF’s immuno-modulatory and anti-inflammatory properties appear to lead to improved outcomes of chronic Hepatitis C, inflammatory bowel disease, DED and have beneficial effects on immune function in the elderly [23,24,25,26,27,28,29]. Taken together this body of work shows the potential for LF in human health. 

Importantly, associations between LF and DED have been reported in humans and animal models. A progressive reduction in the concentration of LF within tear fluid can be observed with ageing and correlates to worsening DED [30,31]. Kawashima et al. previously demonstrated in C57BL/6Cr Slc mice that dietary LF has the potential to suppress inflammation of the lacrimal gland and cornea, as well as oxidative stress; critical components in the pathogenesis of DED [19]. In addition, the capacity of LF as a component of a combination dietary supplement to maintain tear secretion has also been demonstrated in both rats and humans [28]. The wide spanning properties of LF and its well documented safety fuels the potential of LF as a dietary supplement. In fact, LF has already been included in several consumable health products, from yogurts to infant formulae. 

Over the last few decades, extensive studies have led us to develop a deeper understanding of the importance of the complex microbial community that colonised the human gut, known as the gut microbiota. The influence of which is now widely considered a key component to human health and is thought to have a strong influence on the development of the immune system [32]. It would appear that the specific gut microbial species contribute to mucosal and systemic immune homeostasis through induction of the differentiation of several types of immune cells such as regulatory T cells (Treg), which consequently suppresses inflammation via a number of mechanisms, including IL-10 secretion [33,34,35,36]. A potential mechanism of which has been attributed to the intestinal microbial fermentation of dietary fibres, resulting in the production of short-chain fatty acids (SCFA), in particular butyrate which is mainly produced by one of a major bacterial order, Clostridiales [33,36]. Furthermore, previous studies have indicated that SCFA play an active role in the protection against inflammatory diseases [34,35,37,38,39]. SCFA producing bacteria have therefore been proposed as potential probiotics, and by manipulating the gut microbiota, we may be able to enhance gut homeostasis and overall health [36,40].

Because of the association between LF and gut microbiota modulation, and how certain bacterial strains play a role in the suppression of inflammation, we speculated that LF-mediated modulation of the gut microbiota may provide a mechanism for the generation of an anti-inflammatory environment and ultimately protection against DED. Therefore, this study was designed to investigate LF as a potentially useful compound to alleviate the symptoms of DED by approaching the underlying pathogenesis of the disease, in aim of improving the quality of life of sufferers. 

## 2. Results

### 2.1. Lactoferrin Protects against Reduction of Tear Secretion in a Restraint and Desiccating Stress (RDS) Mouse Model of Dry Eye Disease

**Figure 1:**
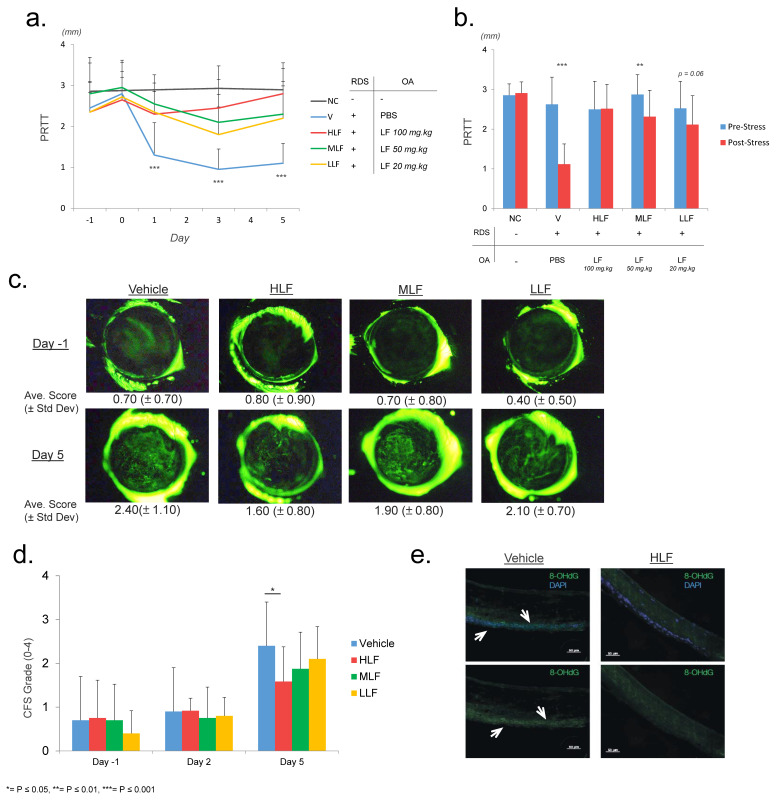
Protection of tear secretion by oral administration of lactoferrin. (**a**). Phenol red thread test (PRTT) results showing the rapid and significant decline in PRTT measurement in V mice after the initial stress event at day 0, whereas the LF administered mice maintained a measurement close to that of pre-stress levels with HLF showing the greatest results. The prolonged hyposecretion in V mice can be easily observed for the remainder of the protocol. (**b**) Bar chart highlighting the pre (day -1 and 0) and post (day 1 to day 5) initial stress event differences in PRTT results. (**c**) Comparison of typical images of corneal fluorescein staining on day 1 and day 5. HLF mice showed a significant reduction in ocular surface damage. (**d**) Average corneal fluorescein staining scores at day 1, 2, and 5. (**e**) Immunofluorescence staining for the oxidative damage marker, 8-OHdG. Positive staining is highlighted by arrows. Positive staining was observed in fewer cells in the corneal epithelium of HLF mice, indicating a reduction in oxidative stress induced damage.

To assess the efficiency of LF as a potential supplement for the treatment of DED, mice were administered an oral solution of either PBS (Vehicle (V)) or LF at 3 different concentrations (Low-LF = 20 mg/kg (LLF), Mid-LF = 50 mg/kg (MLF), High-LF = 100 mg/kg (HLF)). These mice were exposed to RDS to induce DED, as previously described by Nakamura et al. [41]. A 5th group of mice, termed Negative Control (NC), were not exposed to RDS, and did not receive any oral administration (see Table 1 for group overview). We performed a phenol red thread test (PRTT) to analyse the level of mouse tear secretion. Results showed a “resting” level (average of day -1 and day 0) across all 5 mouse groups prior to the initial stress event (NC: 2.9 mm ± 0.3, V: 2.6 mm ± 0.7, LLF: 2.5 mm ± 0.7, MLF: 2.9 mm ± 0.5, HLF: 2.5 mm ± 0.7) (Figure 1a,b). Interestingly, the first PRTT measurement (day 1) after the initial stress event at day 0 indicated that V mice showed a significant decrease to 1.3 mm ± 0.5 relative to NC (2.9 mm ± 0.3) and LF administered mice (HLF: 2.3 mm ± 0.8, MLF: 2.6 ± 0.6, LLF: 2.4 ± 0.7). Furthermore, this low level of tear secretion persisted for the duration of the protocol in V mice (average day 1 to day 5, V: 1.1 mm ± 0.5). Whereas the opposite was true for NC and LF administered mice (average day 1 to day 5, NC: 2.9 ± 0.4, HLF: 2.5 mm ± 0.6, MLF: 2.3 ± 0.7, LLF: 2.1 ± 0.7). The PRTT results of the LF administered groups remained close to that of the initial resting level. HLF mice demonstrated the greatest preservation of tear secretion and showed a significant difference relative to LLF mice at day 5, potentially indicating a dose dependent action of LF (Figure 1a,b). As HLF concentration displayed the greatest protection of tear secretion, the following experiments were performed on HLF mice only. 

To confirm the PRTT results and quantify the effects of RDS on the generation of DED, we performed ocular surface corneal fluorescein staining. This is a commonly performed diagnostic tool for DED in a clinical setting and allows the investigator to observe corneal abrasions and injuries that arise from a loss in tear secretion. Damage was graded by using the Oxford Scale and allowed an observation of a significant increase in V mice by the end of the protocol (Figure 1c,d). Despite the preservation of tear secretion in LF administered mice, a significant increase in corneal damage was observed across all groups between day 1 and day 5. However, the damage was less in HLF, which showed a significantly lower level than V mice on day 5 (average of 1.60 and 2.10, respectively). Furthermore, a significant increase in scoring was not observed until day 5, possibly indicating a gradual progression towards ocular surface damage. 

8-OHdG is indicative of reactive oxygen species (ROS)-induced oxidative stress lesions in both mitochondrial and nuclear DNA. We performed immunofluorescence staining to investigate the presence of 8-OHdG ROS at the ocular surface. At day 5, we observed an obvious increase in 8-OHdG positive staining in V mice, specifically in the epithelium of the cornea (Figure 1e arrows). HLF mice showed considerably less positive staining than vehicle mice; however, isolated areas of positive staining were detected. Our results indicate a protective function of LF against oxidative stress, as previously reported by Kawashima et al. [19]. However, whether this reduction of oxidative stress is a direct result of the administration of LF and its anti-oxidative properties at the ocular surface or a secondary effect stemming from a general reduction in inflammation or of enhanced tear secretion in these mice remains unclear. 

### 2.2. Lactoferrin Induces the Reduction of Inflammatory Cytokines

**Figure 2:**
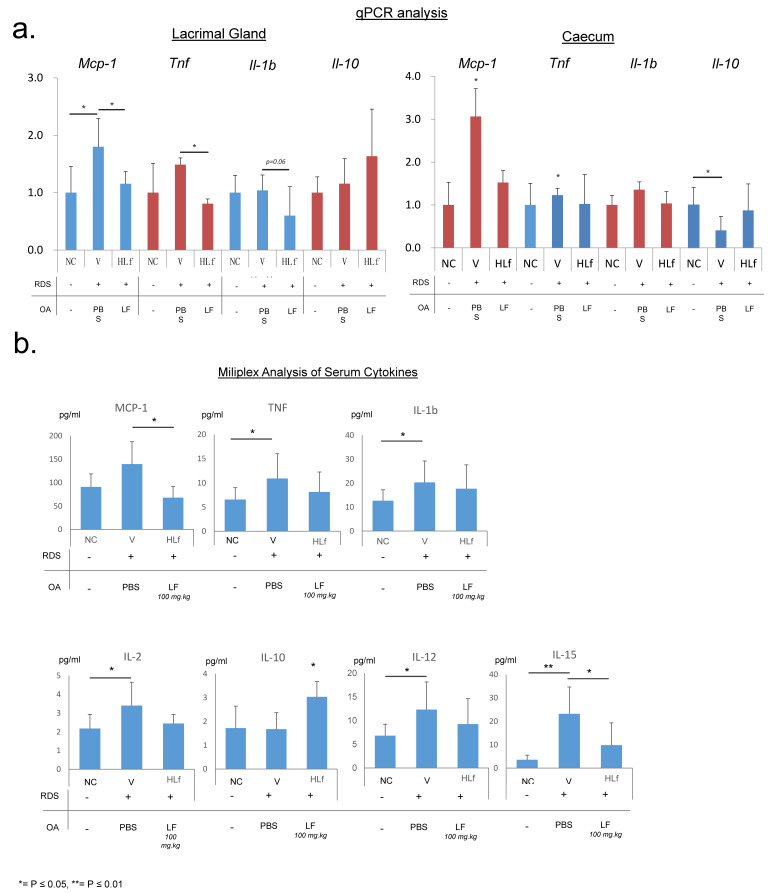
Cytokine analysis. (**a**) qPCR analysis of the expression of *MCP1*, *TNF*, *IL-1b*, and *IL-10* genes in the lacrimal gland and caecum. *β-actin* was used as the housekeeping gene. (**b**) Milliplex assay probing for the concentration of various inflammation-related proteins within the sera, indicating systemic alterations in inflammation. An apparent increase in a level of expression and concentration of cytokines was observed in V mice relative to that observed in HLF mice, suggesting a protective function of LF, leading to a reduction in inflammation.

Due to the pro-inflammatory nature of DED pathogenesis, identifying alterations in the expression of cytokines and chemokines will allow us to determine whether the protective effect of LF on tear secretion is coupled with a reduction in inflammation. Quantitative real-time polymerase chain reaction (qPCR) analysis using RNA extracted from the LG of V mice demonstrated a significant increase in the expression of *MCP-1* and *TNF* relative to HLF, with an approximate 1.75 and 1.5 times increase, respectively (Figure 2a). *IL-1b* showed a decline in HLF mice LG (Figure 2a). These cytokines have previously been implicated in DED pathogenesis [42,43,44,45]. Intriguingly, although only approaching significance, we observed a heightened level of expression of the anti-inflammatory cytokine *IL-10* in the LG of HLF mice, suggesting a potentially protective environment (Figure 2a). 

Due to the potential role of the gut microbiota on intestinal inflammation, cytokine expression within the caecum region of the intestine was also measured (Figure 2a). *MCP-1* relative expression was more than 3 times higher than that of NC and twice that of HLF. *TNF* expression was significantly increased in the caecum of V mice relative to the other groups. Interestingly, although we did not observe an increased expression of *IL-10* in the caecum of HLF mice, *IL-10* level was markedly lower in the caecum of V mice. 

Milliplex assay was performed to confirm the presence and concentration of various cytokines and chemokines. We observed a significant increase in the levels of a number of proinflammatory cytokines in V mouse sera relative to NC, including IL-1b, 2, 6, 12, 15, TNF, and MCP-1 (Figure 2b). Furthermore, in accordance with qPCR results, we observed a significant increase in the concentration of anti-inflammatory cytokine, IL-10, in HLF mice (Figure 2b), which may explain the protective effect of LF on tear secretion in these mice and, interestingly, extensive literature indicates a role for this cytokine in T cell-mediated homeostatic immune regulation. 

### 2.3. Lactoferrin Modulates Gut Microbiome and Induces Short-Chain Fatty Acid Production 

**Figure 3:**
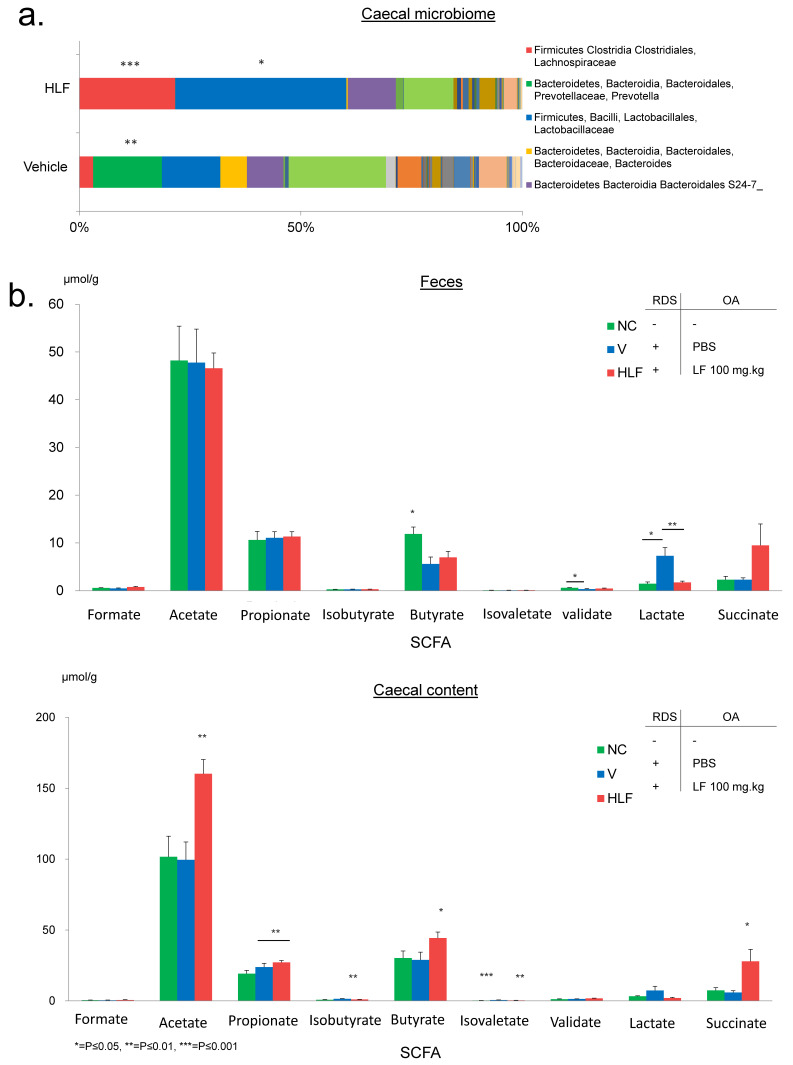
Analysis of the gut microbiome and SCFA composition. (**a**) Next generation sequencing of bacterial genomic DNA from caecal samples of HLF and V mice. Significant increases in a number of commensal bacteria implicated in the production of SCFA were observed in HLF mice. (**b**) Gas chromatography–mass spectrometer analysis of various SCFA and organic acid concentrations in faecal and caecal samples. A significant increase in acetate, propionate, butyrate, isovalerate, and succinate was observed in the caecal content samples of HLF mice relative to V mice. In faecal samples, only butyric acid concentration was significantly higher in NC mice relative to the other groups. A significant difference was observed in valeic acid concentrations between NC and V mice. No significant difference was observed for the remaining SCFA in faecal samples.

The composition of gut microbiome was analysed by using 16S rRNA gene-based amplicon sequencing. Relative abundance of operational taxonomic unit (OTU) can be observed in Figure 3a. We observed significant differences in the composition of bacterial species in the caecum of HLF and V mice. Bacterial order Clostridiales formed over 20% of OTU in HLF and the abundance was significantly higher than that in V mice. Interestingly, this group of bacteria had previously been implicated in the production of SCFA and the induction of regulatory T cells [33,36]. 

To further assess the importance of LF in the modulation of the gut microbiota and the potential subsequent production of SCFA, the concentration of 7 different SCFAs and 2 organic acids was determined by using gas chromatography-mass spectrometry (GC/MS). The concentration of formate, acetate, propionate, isobutyrate, butyrate, isovalerate, valerate, lactate, and succinate was probed for in murine faeces and caecal content. These metabolites are usually found both in faeces and the caecum, with the latter being a major area of fibre fermentation and subsequently absorbed into the blood stream [46,47,48]. SCFA concentration varied a little among the groups in faecal samples, with the exception of butyrate being significantly higher in the faeces of NC mice and an increased concentration of lactate in those of V mice (Figure 3b). However, staggering results were observed in caecal samples. HLF mice displayed a significant increase in acetate, propionate, butyrate, isovalerate, and succinate in the caecum, consistent with the effect of LF on the gut microbiome (Figure 3b). Acetate displayed the greatest increase. Acetate concentration was approximately 100 µmol/g in both NC and V mice, while it rose to an average of 165 µmol/g in HLF mice (Figure 3b).

### 2.4. Vancomycin Abrogates the Effects of Lactoferrin on DED and Significantly Reduces the SCFA Concentrations

**Figure 4:**
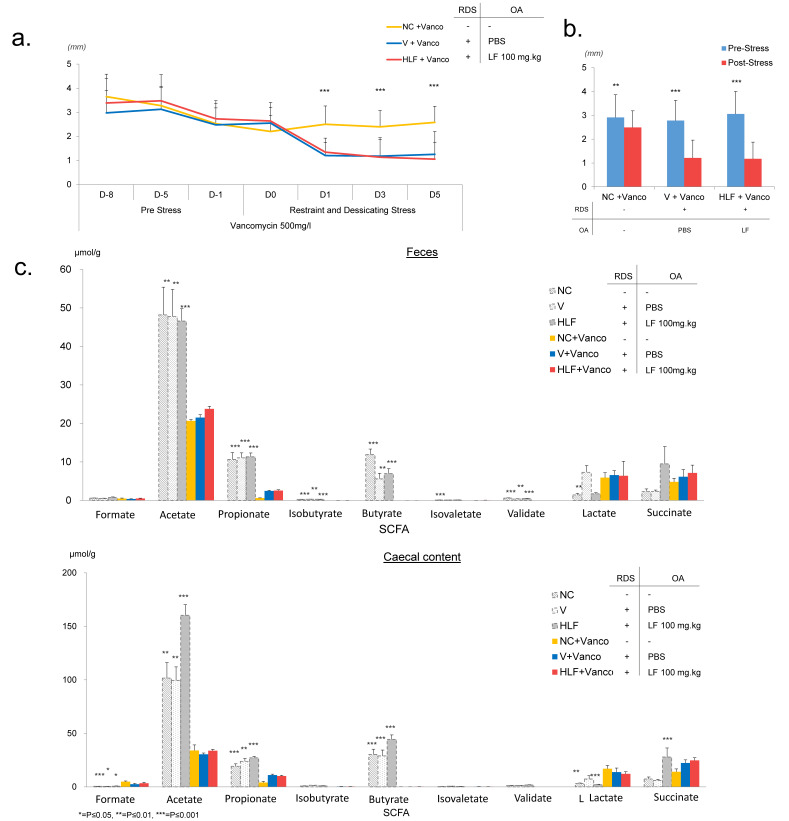
Influence of vancomycin on the effects of lactoferrin. To assess the influence of LF-mediated modulation of the gut microbiota on our PRTT observations, we modified our RDS protocol. Mice were administered the antibiotic, vancomycin, (500 mg/L) in their drinking water to induce gut dysbiosis and, therefore, potentially decrease the effects of LF on the gut microbiota. Mice were preloaded with vancomycin (in drinking water) for 1 week prior to the initial stress. Daily RDS commenced from day 0. Three groups of mice were used, NC + Vanco, V + Vanco, and HLF + Vanco. (**a**) PRTT measurements indicated a gradual decline for all three groups during the preloading phase. Conversely to no vancomycin administered mice in the original PRTT protocol (Figure 1), after the initial stress event, tear secretion further declined in both groups receiving RDS (V + Vanco and HLF + Vanco), indicating that the effects of LF were abrogated by elimination of vancomycin-sensitive bacteria. (**b**) Bar chart demonstrating the difference in PRTT tear secretion level between the beginning and end of vancomycin preloading period (day -8 to day 0). (**c**) Bar chart demonstrating the difference in PRTT measurements between pre-stress period (day -8 to day 0) and post-stress period (day 1 to day 5). GC/MS analysis of SCFA in caecal and faecal samples of vancomycin administered mice compared to that in non-administered mice. A significant decline of all three SCFA analysed in both samples was observed in the three groups of vancomycin administered mice compared to that in the non-vancomycin administered mice, highlighting the influence of the gut microbiota in the production of SCFA.

To determine whether the effects of LF on tear secretion was a direct result of the observed modulation of the gut microbiome and increase in SCFA, we speculated that the administration of the antibiotic, vancomycin, would enable us to eliminate a number of potent SCFA producing bacteria, including *Clostridium* species. This would therefore allow us to determine whether the effect of LF on tear secretion was linked to SCFA concentrations. Three groups of mice were administered a vancomycin solution (500 mg/L) in their drinking water. NC + Vanco group was not exposed to RDS or given oral administration. Whereas, V + Vanco and HLF + Vanco were both exposed to RDS and received an oral administration of PBS or LF (100 mg/kg) respectively (see Table 2 for group overview). We performed PRTT to assess potential changes in tear secretion. Interestingly, all mice displayed a gradual decline in tear secretion in the initial preload phase (day -8 to day -1) (Figure 4a,b). A significant difference between day -8 and day 0 in NC + Vanco and HLF + Vanco mice was observed (Figure 4a,b). However, despite a significant difference between pre-stress and post-stress event in NC + Vanco mice, the trend of a progressively reducing lacrimation stabilised between day 1 and day 5, at an average PRTT measurement of 2.5 mm ± 0.7 (Figure 4a,b). V + Vanco showed a drastic and significant decline in PRTT results after exposure to R&D stress (day -8: 2.9 mm ± 1.0; day 0: 2.6 mm ± 0.9; day 5: 1.25 mm ± 1.0. Pre-Stress average: 2.8 mm ± 0.9; Post-Stress average: 0.8 mm ± 0.8). This was also true in the HLF + Vanco group despite the administration of LF (day -8: 3.4 mm ± 1.0; day 0: 2.6 mm ± 0.6; day 5: 1.1 mm ± 0.7; Pre-Stress average: 3.1 mm ± 1.0; Post-Stress average: 1.2 mm ± 0.7), demonstrating that the protective effect of LF was abrogated by the administration of vancomycin (Figure 4a,b). 

Furthermore, in addition to abrogating the effects of LF on tear secretion, vancomycin also had profound effects on SCFA concentrations in both faecal and caecal samples in all 3 groups. Again, using GC/MS, we observed a significant decline in the concentration of acetate, propionate, butyrate, and valerate across all groups in both faecal and caecal samples (Figure 4c). Butyrate concentration was effectively abrogated in both faecal and caecal samples. However, lactate and formate displayed a minor increase in caecal contents.

## 3. Discussion

In this study, we aimed to investigate the potential of LF for the prevention of DED development and to elucidate the mechanisms underlying LF’s anti-inflammatory ability [19,20,21]. DED is highly inimical to affected people [2]. A range of complex factors contribute to DED pathogenesis, resulting in excessive and chronic inflammation in the LG [1,49], consequently leading to damage of the secretory acini cells and loss of functional tear secretion. In addition, reduced tear fluid at the ocular surface further enhances inflammation, resulting in a self-perpetuating cycle of progressively worsening disease [1,10]. Currently, treatment options for DED are limited; environment alteration, punctual plugs, and artificial tear solutions being the only viable course of action. However, these do not resolve the underlying cause of DED, but only superficially relieve symptoms. This lack of current treatment in addition to a globally ageing population demands attention and research to understand the mechanisms underlying DED and to develop appropriate therapies. 

Our work here focuses on a murine model of DED adapted from Nakamura et al. [41] which utilizes restraint and desiccating stress to induce hypolacrimation. This relatively simple method produces a phenotype in mice mimicking symptoms of the human disease. This reduction in tear secretion is also thought to occur via the induction of excessive reactive oxygen species leading to further impairment of the LG secretory system, and is thus sufficiently representative of DED in humans. However, it remains to be seen whether the results here would be reproducible and applicable to the natural development of DED in patients. We therefore believe that our observations warrant clinical investigation of the impact of LF on DED. 

Here, we demonstrated that the iron binding glycoprotein, LF, prevents the loss of tear secretion in our novel RDS murine model of DED. Furthermore, in accordance with other studies, we saw that LF reduced the expression and concentration of pro-inflammatory mediators, including IL-1b, IL-12, MCP-1, and TNF, in the LG, caecum and in the sera, potentially indicting systemic reduction in inflammation [19,50,51]. These pro-inflammatory mediators have previously been implicated in the pathogenesis of DED [42,43,44,45]. We also detected an increase in the levels of IL-15. Although, IL-15 has not been previously associated with DED pathogenesis, it is a T-cell proliferation factor and, therefore, may potentially play a role in the inflammation observed in our model [52]. Furthermore, an increase in the concentration and expression of IL-10 in HLF mice was observed. IL-10, an essential immunoregulator [53,54], may suppress chronic inflammation that leads to LG destruction. This hypothesis builds upon previous work in our laboratory which similarly demonstrated LF’s ability to protect against age-related inflammation-induced DED [19]. Furthermore, Dogru et al. demonstrated that oral LF maintained tear secretion and ocular surface stability in DED arising from LG destruction by the autoimmune disease, Sjögren’s syndrome [55]. We therefore believe that the protective effect of LF on tear secretion may be due to its anti-inflammatory ability. However, the mechanisms underlying LF’s anti-inflammatory properties have yet to be fully elucidated.

LF is well-known for its ability to sequester ferric iron, leading to the inhibition of bacterial growth, and a capacity to modulate the gut microbiota has been previously demonstrated [15,16]. It is becoming increasingly evident that this population of intestinal bacteria, including probiont and symbiont microbiota, plays a major role in the development of human health and drives immune system development and homeostasis [32,56,57]. In contrast, abnormal disruption in the specific composition known as “dysbiosis” leads to enhanced disease [58,59]. However, evidence indicates that the effects of dysbiosis can be reversed with the restoration of a commensal population [60]. Germ-free mice display increased inflammation, gut hyperplasia, and poor quality mucosal lymphoid generation [32]. However the effects are reversed by colonisation of germ-free mice with bacteria present in murine faeces [61]. Since LF can alter the gut microbiota and can modulate inflammation, we propose that these two properties may be connected in our model. Our results revealed that orally administered LF can alter the specific composition of bacterial species that form the gut microbiota, with a significant increase in the presence of commensal species of Clostridia, which are linked to gut homeostasis and immune development [62,63]. In addition, the expression of a number of pro-inflammatory cytokines within the gut were reduced, which is consistent with LF potentially modulating the gut microbiota. 

Treg cells, a T-cell subpopulation, play a central role in immunosuppression and counteract the inflammatory nature of effector immune cells and their proliferation. These immunoregulatory T-cells can be further divided according to a number of varying phenotypes, including IL-10 producing peripheral T-cells (known as Tr1 cells) as well as CD4+ FOXP3+ tTreg and pTreg cells. Treg cells undergo generation and education within the peripheral lymphoid tissue of the gut, resulting in the development of pTreg cells possessing T-cell receptors restricted to commensal antigens and the development of immunological tolerance [64]. Crucially, these T-cell phenotypes are capable of modulating immune responses to create an environment conducive to anti-inflammatory conditions. This is achieved by a number of methods, with the secretion of IL-10 playing a major role [65,66]. The essential function of these cells is apparent in depleted or abnormal Treg populations, resulting in various spontaneous auto-immune diseases [67]. It is believed that the modulatory effect of the microbiome upon inflammation is derived from a profound influence in the generation of these gut derived peripheral Treg cells, with strong evidence showing their induction by SCFA [68,69]. 

We believe that LF modulates the gut microbiota towards a composition that consequently leads to a significant increase in the concentration of these SCFA in our mouse model. Specific bacterial strains, including commensals such as Clostridia, are potent producers of SCFA [33,36], which could potentially explain the observed increase in HLF mice, but not in NC or V mice. Our results indicating that vancomycin treatment reduced SCFA concentration further support this hypothesis. SCFA can enhance the differentiation of immune-regulatory T-cells via a number of mechanisms. SCFA stimulation of the cell surface receptor GPR43 is thought to lead to endocytosis and the inhibition of histone deacetylase (HDAC) [69], a suppressor of Treg cell expansion [70]. HDAC inhibition by SCFA, especially butyrate, induces epigenetic alterations via stimulating enhanced histone H3 acetylation of genes that lead to the generation of Treg cells such as the conserved non-coding sequences of promoter regions of the *Foxp3* gene [33]. Moreover, HDAC inhibition enhances STAT3 and the mTOR pathway activity, key effectors of a number of regulatory cytokines, including IL10, which acts in a negative feedback loop manner as a crucial mediator of immune regulation and homeostasis [53,68]. Therefore, the potential of LF to indirectly enhance SCFA production via the gut microbiota may lead to an upregulation of immuno-regulatory Treg cells and altered cytokine profile, leading to greater control over systemic inflammation and subsequent protection of LG function, and thus this warrants further investigation. Furthermore, the prevention of the growth of gut bacteria by administration of the antibiotic, vancomycin, not only abrogated the effect of LF on tear secretion, but also led to severely depleted SCFA concentrations, supporting our hypothesis that the gut microbiota may be responsible for the anti-inflammatory effects of LF seen here. 

Extensive research bolsters our argument with multiple studies showing that the presence of SCFA helps reduce inflammation at the gut and beyond [34,35,37,39]. Singh et al. demonstrated that commensally derived butyrate is capable of suppressing colonic inflammation and even carcinogenesis via the GPR109A receptor, which leads to an increased differentiation of Treg and IL10 producing cells [34]. Similarly, Tropette et al. revealed that commensal bacteria increased the levels of both sera and intestinal SCFA, leading to reduced lung inflammation and atopic disease, consistent with our results. Interestingly, they were unable to detect the presence of SCFA within the lung itself, suggesting a wider systemic effect [35]. In fact, Nakamura et al. (2017) demonstrated that SCFAs of the gut ameliorated uveitis in a mouse model by restricting the migration of lymphocytes to the eye [39]. This may provide an explanatory mechanism for the observed effects in our model that warrants further investigation. We believe that our results suggest that LF may rescue tear secretion by inducing a systemic reduction of inflammation, which is subsequently leading to the indirect protection of LG functionality. However, despite the well documented role of the gut microbiota and SCFA in the abrogation of inflammation, it must be taken into to consideration that, because of the complexity of this highly elaborate commensal community, it is unlikely that a single species will be responsible for the observed effects, but the output of the community as a whole is more likely to have a profound impact on the host. Nonetheless, further examination is required to fully understand the specific mechanisms underpinning LF’s relationship with SCFA in protecting lacrimal functionality. 

In conclusion, our results revealed the capacity of LF to alter the gut microbiome and SCFA concentrations, potentially leading to local and systemic anti-inflammation, protecting LG function and tear secretion in mice. This may have benefits over topical treatments by potentially preventing the underlying pathology of LG degradation, rather than solely addressing inflammation at the ocular surface. Nevertheless, it is unclear whether LF would induce similar alterations in the profile of the gut microbiome in humans, and if any such changes in composition would have the same effect as seen here in mice. However, as a variety of bacterial species are known to produce SCFA’s, and the benefits have been shown in both mice and humans, it would not be surprising if the functional impact remained intact. Our results provide interesting insights into the potential mechanisms of LF as an anti-inflammatory agent and warrant further investigation of the protein as a promising dietary supplement that may be useful in the fight against the highly detrimental DED. Because this work is preliminary and based on a model that may not reflect reality in patients, definitive confirmation and subsequent studies are necessary to corroborate our findings. This is particularly true regarding the link to the gut microbiota, and whether the LF dose in question is viable and effective for human patients, therefore, we aim to conduct a clinical trial to further investigate in the near future.

## 4. Materials and Methods

### 4.1. Restraint and Desiccating Model of Dry Eye Disease

Ten-week-old female C57BL/6JJc1 mice were purchased from Nihon Clea, randomly divided into 5 groups (V, HLF, MLF, LLF, and NC) of at least 5 animals each, and housed one group per cage in a controlled environment with a 12-h light/dark cycle and at a constant temperature of 22 ± 1 °C (Table 1). Mice were fed a chow diet and supplied with clean water ad libitum. To prevent travel stress interfering with results, mice were given 1 week to acclimatise to their habitat. 

DED induction protocol lasted for 7 days (day -1 to day 5). V, LLF, MLF, and HLF mice were given a daily oral administration via steel gavage syringe of phosphate buffered saline (PBS) (V mice) or LF (LLF = 20 mg/kg, MLF = 50 mg/kg, HLF = 100 mg/kg) from day -1 until completion of the protocol on day 5. NC mice did not receive any oral administration. V, HLF, MLF, and LLF groups were exposed to a daily 4 h RDS event from day 0 to day 5. A specially developed apparatus was used to induce RDS DED. Mice were placed into small PVC tubing with 25 mm diameter, inducing restraint stress. The tubing was attached to an 18 cm diameter electric fan, providing a constant airflow of 2–4 m/s towards the head of the animal acting as desiccating stress [41]. NC mice were not given any oral administration and did not undergo RDS. 

Tear secretion was determined via PRTT for all 5 groups on day -1, 0, 1, 3, and 5. A 20 mm length thread was gently inserted by using forceps to the lateral canthus of both left and right eyes; any tear secretion induced colour change was then measured in millimetres. To prevent data being distorted by a tear response induced by oral administration or RDS itself, PRTT measurement was performed prior to other procedures. Mice were euthanised and tissues were harvested on day 5.

### 4.2. Corneal Fluorescein Staining (CFS) 

RDS protocol (as described above) was performed with the omission of the PRTT. This was replaced with CFS on day 1, 2, and 5. Prior to CFS mice were anaesthetised by intraperitoneal injection of pentobarbital (300 μL.kg). Two microlitres of 2% fluorescein green was pipetted on to the ocular surface of the anaesthetised mice and observed under a cobalt blue light microscope. Images were taken with at 40x magnification. The degree of ocular surface damage was then graded on the 5-point Oxford Grading Scale (0–4) as previously described [71]. 

### 4.3. Restraint and Desiccating Model of Dry Eye Disease: Vancomycin Procedure

RDS protocol was performed with the notable exception that treated mice were provided drinking water containing vancomycin (500 mg/L) from day -8 to day 5. Three groups of 10 mice each were investigated (NC-Vanco, V-Vanco, and HLF-Vanco) (Table 2). During the pre-RDS phase, additional PRTT was performed at day -8, -5, and -1. The RDS protocol began as usual at day 0. All other variables remained consistent with the RDS protocol. 

### 4.4. Analysis of Inflammatory Cytokines by qPCR

Target tissues were homogenised and RNA extraction was performed with TRIzol (Invitrogen, Waltham, MA, USA)/chloroform method. Nanodrop analysis was performed to determine the concentration of RNA. RNA was diluted with RNAse-free purified water to a concentration of 1000 ng/μL. cDNA was generated from the extracted RNA (1000 ng/μL) using ReverTra Ace^®^ qPCR RT Master Mix (Toyobo Co. Ltd., Osaka, Japan) following the manufacturer’s instructions. 1 μL of RNA was used with 19 μL of qPCR RT Master Mix. cDNA was reverse transcribed by using a thermal cycler on a cycle running at 37 °C for 15 min, 98 °C for 5 min, and finally 4 °C for 5 min. The qPCR mixture included THUNDERBIRD^®^ SYBR^®^ qPCR Mix, Rox reference dye (Toyobo Co. Ltd., Osaka, Japan), and primers. The qPCR was performed according to the manufacturer’s instructions. Expression levels were all relative to those of *β-actin* used as an endogenous control. Experiments were designed and analysed by using Stepone Software v.2.2.2. The qPCR was performed as follows: 95 °C for 60 s, 95 °C for 3 s, 60 °C for 30 s, repeated for 40 cycles, and finally a melting curve was obtained. 

### 4.5. Analysis of Inflammatory Cytokines: Milliplex Assay

1 mL of blood was drawn by using a 22-gauge syringe from the abdominal region of the ascending aorta from each mouse under pentobarbital anaesthesia before euthanasia. Samples were centrifuged, allowing the isolation of sera. Sera were snap frozen in liquid nitrogen and stored at −80 °C. Fifteen microlitres of sera were isolated for Milliplex assay (Millipore Milliplex MAP multiplex panels; Merck Millipore, Burlington, Massachusetts, USA). Analytes probed for included a number of predetermined proinflammatory cytokines and chemokines, including: IL-1b, 2, 10, 12, 15, TNF, and MCP-1. Analysis was performed according to the manufacturer’s instructions.

### 4.6. Immunofluorescence Staining

After euthanasia, the left eye was harvested and embedded in Tissue-Tek^®^ O.C.T. (Sakura, Osaka, Japan) and stored at −80 °C. Samples were sliced by using a cryostat into 5 μm sections at −20 °C and fixed with acetone onto glass slides. Slides were washed in PBS and incubated in 10% blocking agent (donkey serum diluted in 0.3% TritonX-100 in PBS) for 1 h. After blocking, 8-OHdG antibody (Mouse monoclonal IgG1, 1:100. JaICa, Tokyo, Japan) was applied to tissue samples and incubated overnight at 4 °C. Slides were then washed again in PBS and incubated with the secondary antibody (Goat Anti-Mouse Alexafluor 488, 1:200. Invitrogen, Waltham, MA, USA) for 2 h. Slides were washed in PBS and incubated with DAPI for 5 min. Finally, the mounting medium, Permaflour (Thermo Scientific Co., Waltham, MA, USA), was added prior to slide cover. Slides were observed by using Zeiss ApoTome Imager.Z1 (Zeiss, Oberkochen, Germany).

### 4.7. Microbiome Analysis

Bacterial genomic DNA was isolated as described previously [33]. In brief, caecal contents were lyophilised by using VD-800R lyophiliser (TAITEC, Saitama, Japan) for 24 h. Freeze-dried samples were disrupted with 3 mm zirconia/silica beads (BioSpec Products, Bartlesville, OK, USA) by vigorous shaking (1500 rpm for 10 min) using Shake Master Neo (Hirata, Kumamoto, Japan). Caecal samples (10 mg) were suspended with DNA extraction buffer containing 400 mL of 10% (*w*/*v*) SDS/TE (10 mM Tris-HCl, 1 mM EDTA, pH 8.0) solution, 400 mL of phenol/chloroform/isoamyl alcohol (25:24:1), and 200 mL of 3 M sodium acetate. Caecal contents in mixture buffer were further disrupted with 0.1 mm zirconia/silica beads (BioSpec Products, Bartlesville, OK, USA) by vigorous shaking (1500 rpm for 5 min) using Shake Master Neo (Hirata, Kumamoto, Japan). After centrifugation at 15,000× *g* for 30 min at room temperature, bacterial genomic DNA was purified from the extracts by a phenol/chloroform/isoamyl alcohol method. After RNase treatment, the V1–V2 region of the 16S rRNA gene was amplified as described elsewhere [72]. Mixed samples were prepared by pooling approximately equal amounts of PCR amplicons from each sample and subjected to 454 GS JUNIOR (Roche Applied Science, Penzberg, Germany) sequencing according to the manufacturer’s instructions. 16S rRNA gene reads were analysed using QIIME (v1.6): fasta quality files and a mapping file indicating the bar-code sequence corresponding to each sample were used as input. The QIIME pipeline takes this input information and split reads by samples according to the bar code, and classifies all 3900 filter-passed reads of the 16S V1–V2 sequences obtained from each sample into operational taxonomic units on the basis of sequence similarity. It also performs taxonomical classification using the RDP-classifier (v2.5) [73]. The 16S rRNA gene sequences have been deposited in the DDBJ database (http://getentry.ddbj.nig.ac.jp/ accessed on 6 November 2021) under accession number DRA006302.

### 4.8. GC/MS Analysis of Short-Chain Fatty Acid

SCFA concentrations of caecal contents and faeces were determined by gas chromatography–mass spectrometory (GC/MS) [33]. In brief, 10 mg of faecal and caecal samples were disrupted by using 3 mm zirconia/silica beads (BioSpec Products, Bartlesville, Oklahoma, USA) and homogenised with the extraction solution containing 100 mL of internal standard (100 mM crotonic acid), 50 mL HCl, and 200 mL ether. After vigorous shaking using Shake Master Neo (Hirata, Kumamoto, Japan) at 1500 rpm for 10 min, homogenates were centrifuged at 1000× *g* for 10 min and the top ether layer was collected and transferred into new glass vials. Aliquots (80 mL) of the ether extracts were mixed with 16 mL N-tert-butyldimethylsilyl-Nmethyltrifluoroacetamide (MTBSTFA). The vials were sealed tightly, heated at 80 °C for 20 min in a water bath, and left at room temperature for 48 h for derivatization. The derivatised samples were run through a 6890 N Network GC System (Agilent Technologies) equipped with HP-5MS column (0.25 mm 330 m 30.25 mm) and 5973 Network Mass Selective Detector (Agilent Technologies). Pure helium (99.9999%) was used as the carrier gas and delivered at a flow rate of 1.2 mL/min. The head pressure was set at 97 kPa with split 20:1. The inlet and transfer line temperatures were 250 and 260 °C, respectively. The following temperature program was used: 60 °C (3 min), 60–120 °C (5 °C per min), 120–300 °C (20 °C per min). One microliter of each sample was injected with a run time of 30 min. SCFA concentrations were quantified by comparing their peak areas with the standards.

### 4.9. Statistical Analysis

Sample sizes were determined on the basis of pilot experiments and previous experience from similar experiments. We used an F-test to determine whether the data had the same variances. As all the data were determined to be normally distributed, parametric statistics were used throughout. All data were analysed by Student’s *t*-test, one-way ANOVA, or one-way ANOVA with post-hoc Tukey’s test using SPSS software version 25 (IBM, Armonk, New York, NY USA). Statistical significance was established at a threshold of *p* < 0.05. The statistical test used for each experiment is stated in the corresponding figure legend data.

## Figures and Tables

**Table 1:** Overview of the mouse groups in the phenol red thread test protocol.

Mouse Group	Restraint and Desiccating Stress	Oral Administration
Negative Control (NC)	-	-
Vehicle (V)	+	PBS
Low LF (LLF)	+	LF (20 mg/kg)
Mid LF (MLF)	+	LF (50 mg/kg)
High LF (HLF)	+	LF (100 mg/kg)

**Table 2:** Overview of the mouse groups in phenol red thread test with vancomycin.

Mouse Group	Restraint and Desiccating Stress	Oral Administration	Vancomycin
Negative Control (NC) + Vanco	-	-	+
Vehicle (V) + Vanco	+	PBS	+
High Lactoferrin (HLF) + Vanco	+	Lactoferrin (100 mg/kg)	+

## Data Availability

The datasets generated and/or analyzed during the current study are available from the corresponding author on reasonable request.
